# Determination of kinetic bioconcentration in mussels after short term exposure to polycyclic aromatic hydrocarbons

**DOI:** 10.1016/j.heliyon.2017.e00231

**Published:** 2017-01-10

**Authors:** Ledicia Rey-Salgueiro, Elena Martínez-Carballo, Antonio Cid, Jesús Simal-Gándara

**Affiliations:** a Nutrition and Bromatology Group, Analytical and Food Chemistry Department, Faculty of Sciences, University of Vigo, Ourense Campus, E-32004 Ourense, Spain; b Physical Chemistry Department, Faculty of Sciences, University of Vigo, Ourense Campus, E-32004 Ourense, Spain; c REQUIMTE, Departamento de Química, Faculdade de Ciências e Tecnologia, Universidade Nova de Lisboa, Quinta da Torre, 2829-516 Monte de Caparica, Portugal

**Keywords:** Biological Sciences, Food Science

## Abstract

The kinetic bioconcentration of N-heterocyclic aromatic hydrocarbons and polycyclic aromatic hydrocarbons in mussels (*Mytilus galloprovincialis*) after short waterborne exposure was studied. Benzo[a]pyrene (BaP), its analogue azaarene 10-azabenzo[a]pyrene (AzaBaP), and their mixture (Mix), were selected to monitor the changes in water concentrations over three days. Decay of both PAHs concentrations in water after 24 h of waterborne exposure to mussels at levels of 10 and 100 μg/L follows a first order kinetic with half-lives of 4–5 h, with residual levels of PAHs below 7%. While steady-state scenarios are well studied, there is a lack of information of what happens under non-steady-state conditions, the main purpose of our paper. A synergistic bioconcentration of the mixture was found (around 800 in the mix vs. around 400 for individual PAHs at 100 μg/L of waterborne exposure). It could be explained by the following reasons. The most polar AzaBaP does not compete with the most non-polar BaP for the same tissue compartments. Whereas BaP aggregate in hydrophobic areas, AzaBaP can also do in hydrophilic areas. Moreover, a chance for complex formation between them by charge-transfer stabilization mechanisms could make possible a higher bioaccumulation as a mixture. Instead, toxicological results suggest an additive behaviour in the mixture performance, dominated by BaP, which is the key PAH controlling phase I metabolization in mussels, since is approx. three times more toxic. These experiments provide useful indications for a rapid assessment of PAHs kinetic bioconcentration in mussels.

## 1 Introduction

Azaarenes (N-heterocyclic aromatic hydrocarbons) are composed of an aromatic group and a six-membered ring structure, which contain one or more nitrogen atom(s) in place of a carbon atom (Bollag and Kaiser, 1991). They are generated, and released into the marine environment as the result of human activities, by incomplete combustion of fossil fuels, uncontrolled spills, surface runoff, and atmospheric deposition (Chen et al., 2008; Dong et al., 2015; Lintelmann et al., 2010; Yamamoto, 1992). Since they are ubiquitous contaminants in the environment (Sovadinová et al., 2006) their toxicological importance is based on their mutagenic and carcinogenic properties (Mersch-Sundermann et al., 1992; Yamada et al., 2005). Toxicity of homocyclic PAHs and azaarenes increased with increasing number of rings. Some azaarenes, particularly four- and five-ring compounds, express higher mutagenic and carcinogenic activity in comparison to their corresponding PAHs (Sovadinová et al., 2006). Sovadinová et al. (2006) showed that the azaarenes were significantly more cytotoxic and stronger inducers of AhR than their homocyclic compounds. They have been shown to bioaccumulate in aquatic organisms and acute toxic effects have been reported in several fish species, but only in a few aquatic invertebrates and algae (Kraak et al., 1997a; Kraak et al., 1997b). PAHs can enhance the intracellular generation of reactive oxygen species (ROS) with subsequent oxidative damage to macromolecules. Benzo[a]pyrene (BaP) is one of the most common and studied PAHs in the aquatic environments due to its carcinogenic and mutagenic properties (ATDRS, 1995). 10-azabenzo[a]pyrene (AzaBaP) is an analogue of BaP with a nitrogen atom at position-10, which so far has not yet been studied in comparison to BaP. The comparative performance is of key importance when oil spills and disasters take place in coastal areas to understand the environmental damage.

Spain is the second largest producer of mussels in the world and the top European producer, with an output of 300,000 tons per year (*M. galloprovincialis*), of which 99% is produced in Galicia (NW Spain), where mussels are an important socio-economical resource (http://www.reproseed.eu/Species/Blue-mussels). Mussels are filter-feeding bivalves that are used as sentinel species in environmental monitoring due to their physiology, behaviour and their broad study in cellular, genetic and biochemical level (Dailianis, 2011). Their ability to accumulate and tolerate high concentrations of many organic pollutants including PAHs, reinforce the role of mussels as bioindicators of the marine environment (Vidal-Liñán et al., 2010). The aim of the present work was to evaluate the waterborne exposure of mussels to BaP and AzaBaP (separately and in mixture) and their kinetic bioconcentration in mussels (*M. galloprovincialis*). In order to estimate and compare the toxic potential of BaP and AzaBaP or its mixture in mussels, induction equivalence factors (IEFs) were calculated.

## 2 Materials and methods

### 2.1 Chemicals, solvents and reagents

Benzo[a]pyrene (BaP) and 10-Azabenzo[a]pyrene (AzaBaP) were purchased from Sigma Aldrich (Madrid, Spain). SPE Silica cartridges were obtained from Phenomenex (Madrid, Spain). The solvents used (all purchased in HPLC-gradient grade) including ethyl acetate, n-hexane, water and acetonitrile (ACN) were from Sigma–Aldrich (Madrid, Spain). All the other chemicals were of analytical grade, and were purchased from local companies.

### 2.2 Mussels exposure experiments

**Fig. 1:**
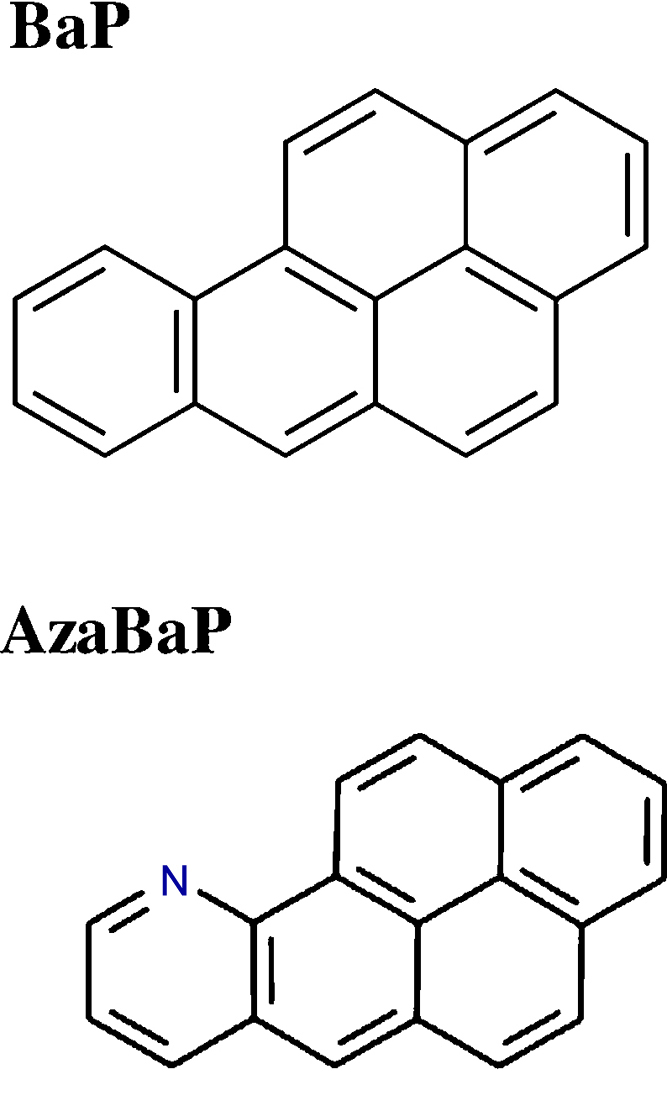
Molecular structure of the studied PAHs: Benzo[a]pyrene (BaP; CAS Number: 50-32-8; M.W.= 252.31 g/mol; Log K_ow_ = 5.99 at 25 °C), and 10-Azabenzo[a]pyrene (AzaBaP; CAS Number: 24407-49-6; M.W.= 253.30 g/mol; Log K_ow_ = 5.08 at 25 °C). K_ow_ were available at ChemSpider; for Log K_ow_ > 5.0, there is a high potential for bioaccumulation.

Mussels (*M. galloprovincialis*) used in this study were caught in sites from local populations in the Ría de Vigo (Galicia, NW Spain) considered to be away from obvious inputs, in May 2014. Prior to the experiments, animals (N = 98, average tissue wet weight of 3.10 ± 0.33 g) were randomly distributed (n = 14) in experimental aquaria (30 L) and subjected to an acclimation period of 1week before the addition of the contaminants. Mussels were kept in aerated seawater at 36‰ of salinity and 15 °C, and under an 8:16 h (light:dark) photoperiod. After the acclimatization period, mussels were exposed to nominal water concentrations of 10 and 100 μg/L of BaP and AzaBaP individually and in two mixtures (5.0 μg/L BaP + 5.0 μg/L AzaBaP, 50 μg/L BaP + 50 μg/L AzaBaP) for 3 days. These conditions were selected since they were found in seawater after an oil spill and subsequent dispersion in the water column (Zuijdgeest and Huettel, 2012). BaP and AzaBaP stock solutions ranging between 0.30 and 3.0 g/L were prepared in acetone and were administered directly into the aquaria. Fig. 1 shows the structures and relevant information for both contaminants under study. A solvent control group (acetone alone) was also used. The solvent concentration in the experimental aquariums was 0.003%. For the study of the possible changes in water treatments, water samples were collected, in triplicate, at 0, 8, 16 and 24 h after the addition of the contaminants from all aquaria. At 48 h was also checked that decay, after the spike at 24 h, was at the same level than after the first 24 h exposure.

In the case of mussel kinetic bioconcentration, experiments were conducted under static conditions and the water was renewed daily, followed by the addition of solvent or contaminant solutions to each one of the treatment groups. The animals were not fed during the assay. Four mussels were harvested from each aquarium after 24 and 48 h, and six after 72 h from the beginning of the experiment. Then, they were stored at −80 °C until further use. The amount of seawater in the aquaria was adjusted proportionally after mussel collection at 24 and 48 h to maintain constant the ratio of number of mussels and volume of sea water (0.46). All experimental treatments as well as the solvent control were duplicated with two independent aquaria for each assay. No mortality was observed in either control or treated group.

All experimental animal procedures were carried out in accordance to the European Union Council (2010/63/EU), and the Spanish Government (RD 55/2013) legal requirements.

### 2.3 PAHs determination

#### 2.3.1 HPLC-FD separation and detection conditions

**Fig. 2:**
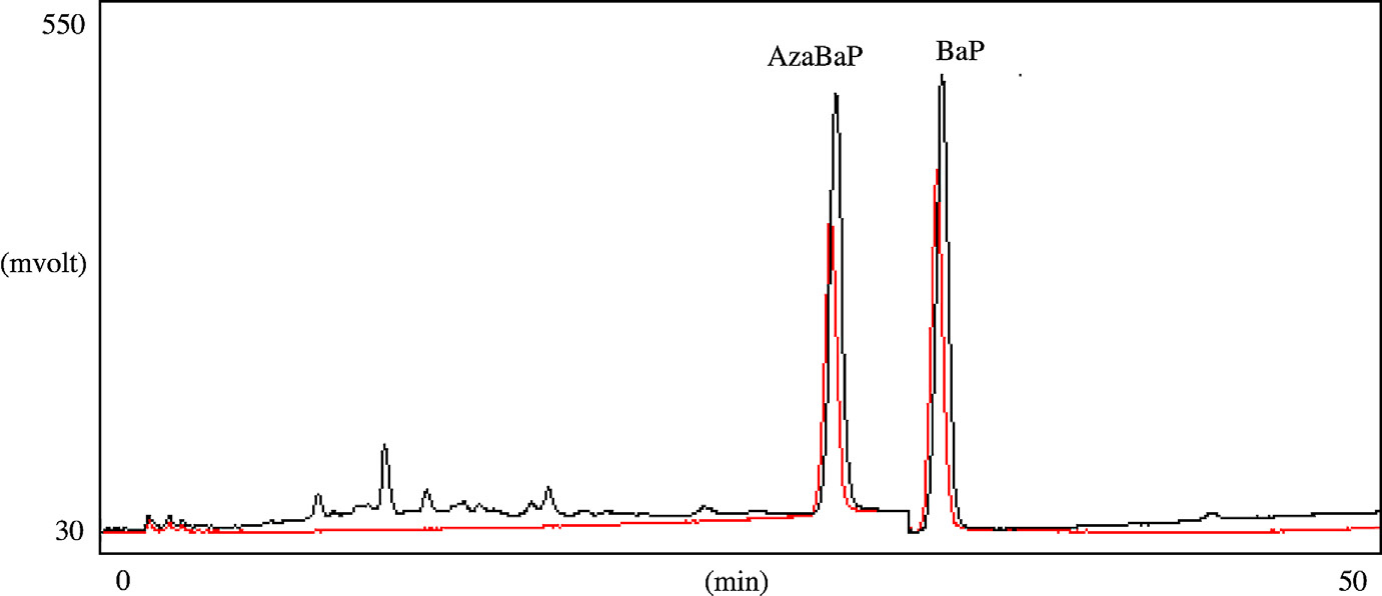
LC–FD chromatograms: standard chromatogram at 2 μg/L (black chromatographic trace), and a mussel extract after waterborne exposure to a mixture of 50 μg/L of AzaBaP and BaP (100 μg/L of total exposure) for 72 h (red trace).

The authors (Rey-Salgueiro et al., 2009; Rey-Salgueiro et al., 2011; Yebra-Pimentel et al., 2013) already published liquid chromatographic conditions. In short, separations were performed with a 25 cm × 4.6 mm (length × i.d.), 5.0 μm particle, Supelcosil LC-PAH column from Supelco (Madrid, Spain). Column temperature was kept at 32 °C. Mobile phases were acetonitrile (A phase) and water (B phase). The used gradient was: 75% A (2 min), changing to 90% A in 43 min, changing to 100% A in 0.1 min, hold for 5 min, changing to 70% A in 0.10 min, and then hold for 10 min (60 min total analysis time). Extract injection was set to 50 μL, and the column flow rate was 1.0 mL/min. Excitation and emission wavelengths were, respectively, set at 296 and 406 for BaP, and 285 and 420 for AzaBaP. A chromatogram of a mussel sample after waterborne exposure to a mixture of BaP and AzaBaP is shown in Fig. 2.

#### 2.3.2 Water BaP and AzaBaP extraction

The extraction of PAHs from water was described by Cheikyula et al. (2008) with minor modifications. In brief, to reach 100% recoveries, water (30 mL) was subjected to three consecutive extractions in a circular shaker with 10 mL n-hexane (15 min each). The organic layers were separated and dehydrated with anhydrous sodium sulphate. Then, the n-hexane extracts were evaporated to dryness under a gentle steam of nitrogen in a Concentration Workstation; the residue was re-dissolved in 1.0 mL of acetonitrile for HPLC analysis.

#### 2.3.3 Mussel BaP and AzaBaP extraction

The extraction of PAHs in bivalves was previously reported (Rey-Salgueiro et al., 2009). In brief, lyophilized mussels (0.25 g) were subjected to three consecutive ultrasound-assisted solvent extractions (UASE) with 10 mL n-hexane:ethyl acetate (3:1) for 10 min each. The separated liquid phases were centrifuged (1000 rpm/min for 5 min), and evaporated until dryness. The residue was re-dissolved in 5.0 mL of extraction mixture and cleaned up with Sep-pack silica plus cartridges, loading additional 10 mL n-hexane:ethyl acetate (1:1). Then, the extract was evaporated to dryness and filled up to a final volume of 1.0 mL with acetonitrile for HPLC analysis. Pooled samples were used (2 mussels per pool).

### 2.4 Statistical analysis

Analysis of variance (ANOVA) and Fisher’s least significant difference (LSD) tests.

The statistical analysis was performed with the statistical software package Statgraphics Plus v. 5.1 (Manugistics, Rockville, MD, USA). Significant differences in the parameter measured (total PAHs) amongst the different factor levels of treatment and time (treatment: control, and 10 and 100 μg/L of single and mixture PAHs; and time: 24, 48 and 72 h) were detected by two-way analysis of variance (ANOVA) at the 95.0% confidence level. A Fisher’s least significant difference (LSD) test, at a 95% confidence level, was used to detect interactions amongst the treatments when significant differences were found by the ANOVA tests. Significant differences at the 95% probability level were found when the confidence interval for the mean is not overlapping amongst the tested treatments.

## 3 Results and discussion

### 3.1 Overall method performance

Method performance was assessed by evaluating the following quality parameters of the method: recovery values, reproducibility, linearity and limits of detection and quantification (LODs and LOQs). Accuracy of the method was evaluated using spiked mussels at 0.14 and 0.56 μg/Kg f.m. of BaP and AzaBaP. The recoveries obtained ranged between 89 and 104% with RSD (%) lower than 7.0% for both tested contaminants. The selected method was robust enough to quantify BaP and AzaBaP in mussels. External standard calibration was chosen to quantify analyte values by LC-FD technique using six calibration standards with concentrations ranged from 0.070 to 1.1 μg/Kg f.m. In order to statistically validate the regression analysis, the linearity was verified by the Mandel fitting test (P = 99%) (Mandel, 1964). A good linearity was obtained for each compound evaluated with correlation coefficients over 0.999 and 0.998 for BaP and AzaBaP, respectively. LODs and LOQs were evaluated based on the noise obtained with the analysis of unfortified blank samples (n = 4). They were calculated following the signal-to-noise criteria (S/N = 3 and S/N = 10 for LODs and LOQs, respectively). The estimated LODs and LOQs were 0.020 and 0.070 μg/Kg f.m., respectively, for both tested contaminants.

### 3.2 Findings on mussels' exposure to BaP and AzaBaP

This is the first study based on the comparison of the changes in concentrations of BaP and AzaBaP in water. This study takes also in consideration the kinetic bioconcentration observed in mussels (*M. galloprovincialis*) after their waterborne exposure. In order to know the real concentrations of BaP and AzaBaP in the water of experimental aquaria, their levels were measured at 0, 8, 16 and 24 h after the addition. After the addition of the contaminants, the real concentrations of BaP and AzaBaP in water samples were similar to nominal concentrations (Table 1). There was a fall in concentrations of both chemicals over time in each one of the treatment groups (in single and in mixture exposure). Changes in the concentrations of both PAHs and their mixtures in water after 24 h exposure at 10 and 100 μg/L follows a first order kinetic with half-lives of 4–5 h (Table 1). After 24 h, and before the addition of fresh contaminant to the aquaria, the concentrations of PAHs still present in each one of the treatment groups were always below 7% (Table 1). Mussels concentrated the remaining amount of PAHs (Table 1). A small fraction could be also adsorbed onto aquarium walls, mussel shells or colloidal material in water suspension, or degraded by biotransformation or photolysis (Suteau et al., 1988).

**Table 1:** First order kinetic with half-lives and BaP and AzaBaP concentrations in water and in mussels after waterborne exposure at 10 and 100 μg/L along time.

PAHs		Treatment (μg/L)	Half lives in water		Concentrations in water		Relative concentration in water (%PAHs ± RSD)	Concentrations in mussels		
			K (h^−1^)	t_1/2_ (h)	(μg/L water ± s.d.)			(mg/Kg mussel f. m. ± s.d.)		
					0 h	24 h	24 h	24 h	48 h	72 h
Control		0			n.d.	n.d.	0	0.04 ± 0.21^a^	0.02 ± 0.17^a^	0.06 ± 0.29^a^
BaP		10	0.17	4.0	11.5 ± 0.9	0.15 ± 0.01	1.30 ± 0.09	1.9 ± 0.3^b^	3.77 ± 0.03^b^	5 ± 2^b^
		100	0.14	4.9	97 ± 6	3.2 ± 0.1	3.3 ± 0.1	15.7 ± 0.1^c^	20 ± 6^c^	34 ± 5^c^
AzaBaP		10	0.14	5.1	9.8 ± 0.3	0.38 ± 0.04	3.87 ± 0.04	1.7 ± 0.3^b^	3 ± 1^b^	5.8 ± 0.2^b^
		100	0.17	4.2	102 ± 8	1.14 ± 0.05	1.12 ± 0.05	14 ± 3^c^	23 ± 4^c^	40 ± 8^c^
Mix	Total*	10			9.3 ± 0.2	0.40 ± 0.05	4.3 ± 0.5	2.11 ± 0.03^b^	4.6 ± 0. 7^b^	7 ± 1^b^
		100			88 ± 5	0.80 ± 0.04	0.91 ± 0.05	18 ± 1^c^	37 ± 4^d^	70 ± 8^d^
	BaP	5	0.16	4.3	4.52 ± 0.06	0.090 ± 0.006	1.9 ± 0.1	1.07 ± 0.04	2.4 ± 0.2	3.9 ± 0.2
		50	0.22	3.1	48 ± 2	0.15 ± 0.01	0.31 ± 0.02	9.4 ± 0.1	19 ± 2	37 ± 5
	AzaBaP	5	0.12	5.6	4.77 ± 0.09	0.32 ± 0.04	6.6 ± 0.9	1.03 ± 0.07	2.1 ± 0.5	2.8 ± 0.5
		50	0.15	4.6	40 ± 3	0.65 ± 0.03	1.64 ± 0.08	8.6 ± 1.5	18.14 ± 2.00	33 ± 5

n.d.: not detected.

*Total: total concentrations in the mixture result from the addition of individual concentrations of BaP and AzaBaP.

a, b, c, d: column-wise significant differences (p < 0.05) for concentrations in mussels at different exposure times (24, 48, or 72 h).

**Fig. 3:**
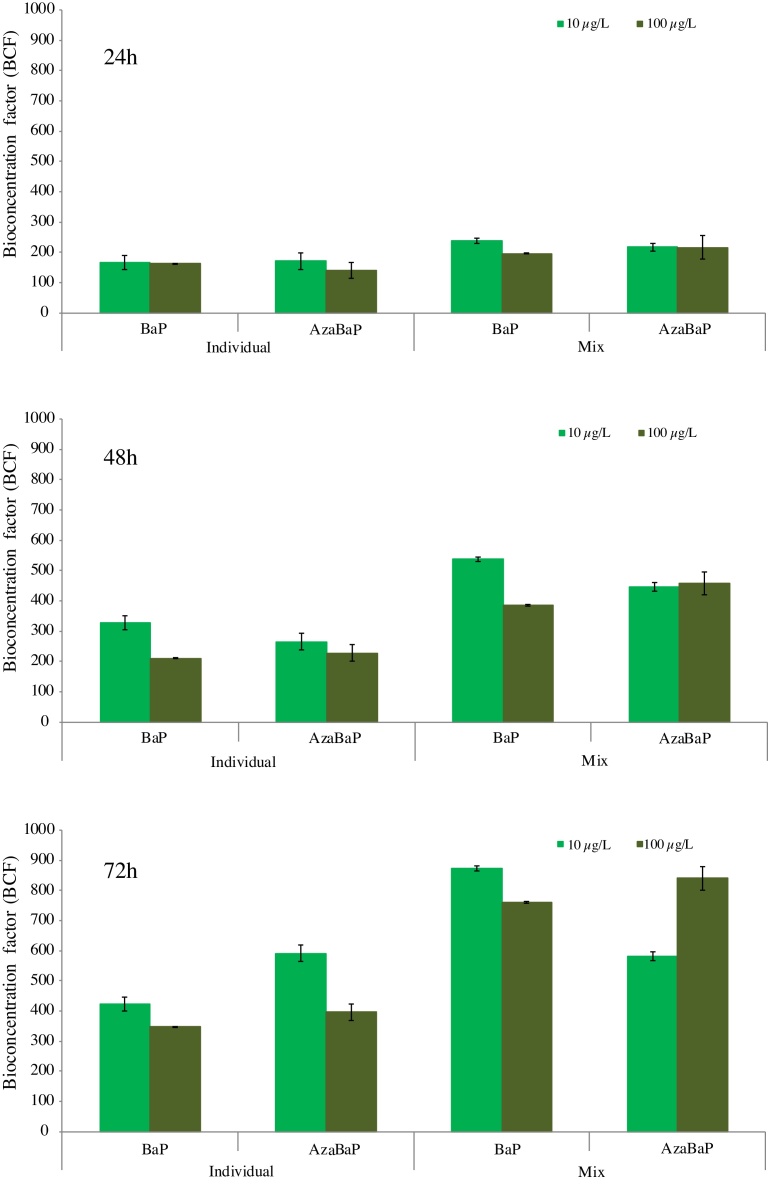
Kinetic bioconcentration factors (BCF_K_) of BaP and AzaBaP in mussels after 24, 48 and 72 h of waterborne exposure to 10 and 100 μg/L (both separately dosed and as a mixture).

The kinetic bioconcentration factor (BCF_K_) of a chemical is the ratio of its concentration in the organism and in water (Opperhuizen, 1991) under non steady-state conditions. The BCF_K_ of BaP and AzaBaP at 24 h were calculated considering the concentration in mussels (body whole) at 24 h and the levels in water at time 0 h (Fig. 3). The accumulation of PAHs in mussels was increasing with the exposure time. A similar kinetic bioconcentration has been found for BaP and AzaBaP at 48 h and 72 h in all experiments, comparing the concentration found in mussel with the concentration added in water 24 h before. Higher BCF_K_ were detected for AzaBaP at 72 h of exposure. Regarding to the exposure level, differences were greater at 72 h in individual experiments with the lowest accumulation at 100 μg/L *νs*. 10 μg/L, probably due to the saturation of mussels’ tissues. Mussels exposed to BaP and AzaBaP as a mixture (MIX) showed a higher accumulation than those exposed to the individual contaminants.

Significant differences in the parameter measured (total PAHs) amongst the different factor levels (treatment: control, and 10 and 100 μg/L of single and mixture PAHs; and time: 24, 48 and 72 h) were used to detect interactions amongst the variables treatment and time by 2-way ANOVA (Table 1). The PAHs kinetic bioconcentration in mussels is clearly different at the waterborne exposure of 100 μg/L, but also the mixture exposure gives significantly higher kinetic bioconcentration with time than those of independent PAHs; the highest differences were found after 72 h exposure. When dividing PAHs variance between the two components, treatment accounts for the 68% whereas time does for the 25%, leaving a 7.2% of unexplained variance or error. It is clear that the differences in PAHs kinetic bioconcentration are higher at 100 μg/L, with exposure time and for the PAHs mix, as also showed the BCF_K_. The synergistic kinetic bioconcentration of the PAHs mixture could potentially be explained by the following reasons. The most polar AzaBaP does not compete with the most non-polar BaP for the same tissue compartments (Huckins et al., 1997). This fact increases the chances for overall kinetic bioconcentration, since −whereas BaP aggregate in hydrophobic areas- AzaBaP can also do in hydrophilic areas (Carrell et al., 1997). There would be also a chance for a kind of complex formation between them by charge-transfer stabilization mechanisms (Wentworth and Chen, 1963) that could make possible a higher kinetic bioconcentration as a mixture.

In order to estimate and compare the toxic potential of BaP and AzaBaP or the mixture of these pollutants in mussels, induction equivalence factors (IEFs) were calculated according to Fent and Bätscher (2000) in relation to a reference substance, Dibenzo[a, h]anthracene. They used the concept of IEF and induction equivalency (IEQ), analogous to toxic equivalence factor (TEF) and equivalence toxic effects (TEQ). Mussel −related IEFs were estimated for single compounds BaP and AzaBaP and as a mixture based on EC50 values of their EROD activities. No EC50 values of EROD activities for BaP and AzaBaP in mussels were reported, so data obtained from fish cell systems were used in the present study (Fent and Bätscher, 2000; Jung et al., 2001). It is known that the rates of the metabolism in tissues of most invertebrates such as mollusc bivalves are lower than in vertebrates as fish (Neff, 2002). The present study could be a first approximation to known the potential effect of these contaminants in mussels in the most restrictive case.

**Fig. 4:**
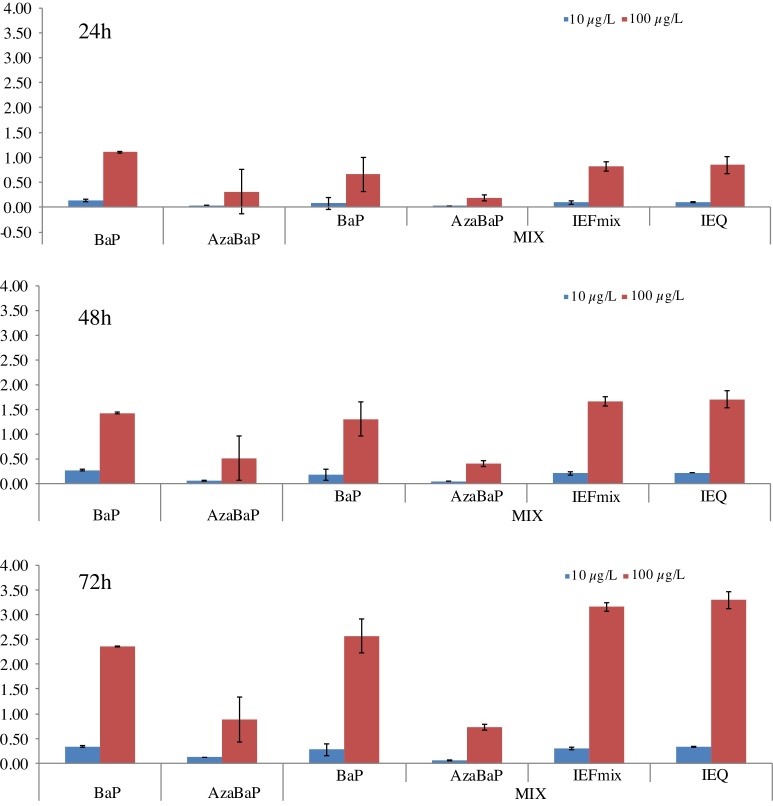
Induction equivalent factors (IEFs) and induction equivalency (IEQ) of BaP and AzaBaP in mussels after 24, 48 and 72 h of waterborne exposure to 10 and 100 μg/L (both separately dosed and as a mixture).

The average EC50 values of both single compounds (BaP and AzaBaP) and the value of the reference substance DBA (Dibenzo[*a,h*]anthracene) reported by different authors (Fent and Bätscher, 2000; Jung et al., 2001) were: EC50_DBA(ref)_ = 1.4 E-8 M; EC50_BaP_ = 2.0 E-7 M and EC50_AzaBaP_ = 6.4 E-7 M. Based on these EC50 values of the single contaminants, a theoretical EC50_Mix(calc)_ was calculated as: EC50_Mix(calc)_ = (2 × EC50_BaP_ × EC50_AzaBaP_)/(EC50_BaP_ + EC50_AzaBaP_) = 3.1 E-7 M (Fent and Bätscher, 2000). The IEFs of individual PAH were calculated as IEF = (EC50_ref_/EC50_PAH_) × C_PAH,_ whereas from EC50_ref_ to the reference PAH, EC50_PAH_ to the EC50 values of individual PAHs and C_PAH_ to the concentration of that individual contaminant detected in mussels. The IEFs calculated for both PAHs at different concentration levels were increasing with the exposure time, and they were higher at 100 μg/L (Fig. 4). IEFs were higher for BaP than AzaBaP (Fig. 4**)**. IEFs for individual BaP were similar to those obtained for BaP in a mixture (Fig. 4). This can be explained because BaP potencies in the mixture (determined as EROD activity) dominates the relative CYP1A induction. Since EC50 for BaP is three times lower than for AzaBaP, BaP could be the key PAH controlling phase I metabolization in marine organism predators, like humans, and therefore in their associated mutagenicity.

Fent and Bätscher (2000) defined IEQ as: IEQ = Σ(C_PAH_ x IEF_PAH_), where C_PAH_ is the concentration of the selected PAH in the mixture. In the present study the IEQ were 0.1, 0.22 and 0.34 for the exposure to 10 μg/L at 24, 48 and 72 h, respectively, and 0.85, 1.7 and 3.2 for the exposure to 100 μg/L at 24, 48 and 72 h, respectively. According to these authors, if determined EC50 (mix) is equal to the calculated EC50 (mix), the mixture shows an additive behaviour. Based on this fact, since IEFs (mix) are equal to IEQ (mix), the results suggest an additive behaviour in the mixture performance.

## 4 Conclusions

The obtained results showed the change in water concentrations of both PAHs and their mixtures after 24 h of waterborne exposure to mussels at levels of 10 and 100 μg/L to levels below 7% with half-lives of 4–5 h. At the same time, the kinetic bioconcentration factors (BCF_K_) were estimated. The mixture of both selected contaminants showed a higher kinetic bioconcentration than separately, doubling the values obtained with the experiments performed separately. Whereas steady-state scenarios are well studied, there is a lack of information of what happens under non-steady-state conditions, the main purpose of our paper. The main findings in this situation were:

1 A synergistic bioconcentration of the mixture was found. It could potentially be explained by the following reasons. The most polar AzaBaP does not compete with the most non-polar BaP for the same tissue compartments. This fact increases the chances for overall bioaccumulation, since – whereas BaP aggregate in hydrophobic areas – AzaBaP can also do in hydrophilic areas. There would be also a chance for a kind of complex formation between them by charge-transfer stabilization mechanisms that could make possible a higher bioaccumulation as a mixture.
2 The toxicological results suggest an additive behaviour in the mixture performance. Anyway, BaP dominated the relative CYP1A induction potencies in the mixture, since EC50 for BaP is three times lower than for AzaBaP.

## Declarations

### Author contribution statement

Ledicia Rey-Salgueiro, Elena Martínez-Carballo, Antonio Cid, Jesús Simal- Gándara: Conceived and designed the experiments; Performed the experiments; Analyzed and interpreted the data; Contributed reagents, materials, analysis tools or data; Wrote the paper.

### Competing interest statement

The authors declare no conflict of interest.

### Funding statement

This work was supported by EU FEDER funds. Antonio Cid was supported by FCT (Portugal).

### Additional information

No additional information is available for this paper.
